# Plant metabolomics: applications and challenges in the era of multi-omics big data

**DOI:** 10.1007/s42994-024-00194-0

**Published:** 2025-01-23

**Authors:** Yingchen Hao, Zhonghui Zhang, Enxi Luo, Jun Yang, Shouchuang Wang

**Affiliations:** 1 National Key Laboratory for Tropical Crop Breeding, School of Breeding and Multiplication (Sanya Institute of Breeding and Multiplication), Hainan University, Sanya, 572025 China; 2 National Key Laboratory for Tropical Crop Breeding, College of Tropical Agriculture and Forestry, Hainan University, Sanya, 572025 China; 3 Yazhouwan National Laboratory, Sanya, 572025 China

**Keywords:** Metabolomics, Mass spectrometry, Multi-omics, Metabolic pathways, Metabolic regulation

## Abstract

Plant metabolites are crucial for the growth, development, environmental adaptation, and nutritional quality of plants. Plant metabolomics, a key branch of systems biology, involves the comprehensive analysis and interpretation of the composition, variation, and functions of these metabolites. Advances in technology have transformed plant metabolomics into a sophisticated process involving sample collection, metabolite extraction, high-throughput analysis, data processing, and multidimensional statistical analysis. In today’s era of big data, the field is witnessing an explosion in data acquisition, offering insight into the complexity and dynamics of plant metabolism. Moreover, multiple omics strategies can be integrated to reveal interactions and regulatory networks across different molecular levels, deepening our understanding of plant biological processes. In this review, we highlight recent advances and challenges in plant metabolomics, emphasizing the roles for this technique in improving crop varieties, enhancing nutritional value, and increasing stress resistance. We also explore the scientific foundations of plant metabolomics and its applications in medicine, and ecological conservation.

## Introduction

Plant metabolites encompass all the small molecules within plant cells that are vital for growth, development, environmental adaptation, and defense (Weng et al. 2021). Primary metabolites, including sugars, lipids, and amino acids, are essential for fundamental physiological functions in plants, such as photosynthesis, respiration, and energy metabolism (Wang et al. 2019). Secondary metabolites (also known as specialized metabolites), such as alkaloids, flavonoids, and terpenoids, are crucial for mediating plant–environment interactions, including defense against diseases and pests, as well as adaptation to various abiotic stresses (Dong and Lin 2021). These compounds not only influence plant survival and reproduction, but also affect the balance of the ecosystem processes and agricultural productivity. Information about plant metabolites offers valuable insights into how plants respond to environmental changes, guiding advances in plant breeding and biotechnology.

Plant metabolites are important for several reasons. First, they are fundamental for plant growth and development, guiding processes from seed germination to plant maturation and reproduction (Weng et al. 2021). Second, they allow plants to adapt to environmental changes, such as by regulating antioxidant biosynthesis in response to stress (Liu et al. 2022). Finally, many secondary metabolites have bioactive properties, offering protection to plants while also providing valuable medicinal and nutritional resources for humans (Li et al. 2022b; Zhu et al. 2020). The study of plant metabolites is motivated by several factors. Advances in molecular and systems biology allow researchers to explore the complexity of plant life through metabolomics. This research supports the development of improved crop varieties with enhanced nutritional content and stress resilience, while also offering a reservoir of compounds for the potential discovery of novel drugs.

Metabolomics is a rapidly evolving field within systems biology that aims to comprehensively analyze the small-molecule metabolites in biological samples. Research on plant metabolites is rapidly progressing. With the application of high-throughput analytical techniques, such as mass spectrometry (MS) and nuclear magnetic resonance (NMR), researchers can more comprehensively analyze and identify plant metabolites (Moco et al. 2007). Furthermore, combining genomics, transcriptomics, and proteomics data with bioinformatics tools has facilitated metabolite identification and the reconstruction of metabolic pathways (Ding et al. 2023; VenegasMolina et al. 2021). Recently developed single-cell metabolomics and spatial metabolomics techniques (e.g., mass spectrometry imaging) allow for more refined detection of metabolites at the cellular or tissue level and provide insights into the distribution of metabolites (Heinemann and Zenobi 2011; Pandian et al. 2023).

Despite many challenges, technological advancements and innovative methods are opening new avenues for understanding plant metabolism. These developments can reveal metabolic regulatory mechanisms and provide a scientific foundation for crop improvement, drug development, and ecological conservation. Moving forward, progress in plant metabolomics will depend on interdisciplinary collaboration, integrating expertise from biology, chemistry, physics, and computer science. In this review, we highlight recent advances in plant metabolite research, examine the roles of metabolomics in plant science research, and explore future directions. We aim to offer valuable insights and inspiration for further studies in plant metabolomics.

## Plant metabolite content as a dynamic, precise plant phenotype

Nearly all life processes in organisms occur at the metabolic level, driven by biochemical reactions regulated by genes and environmental interactions. Metabolites function as executors of gene functions, making them critical to the survival strategies of organisms, not only as mediators of energy and material exchange, but also as important signaling molecules in response to environmental changes (Wang et al. 2019). Most plant metabolites, which include all intermediates and products of metabolic pathways, are small molecules with molecular weights below 2000 Da (Fernie et al. 2004). At the cellular level, the regulation of these pathways directly affects gene expression and protein synthesis, ultimately shaping a plant’s adaptive responses to environmental stimuli (Zhang and Fernie 2021). Environmental changes, including changes in nutrient availability, temperature, and light intensity, affect the concentration and distribution of metabolites, thereby regulating gene activity. For instance, when an organism faces starvation, it activates specific metabolic pathways to promote fatty acid degradation for energy production. This process involves a series of dynamic changes in the concentrations of metabolites, such as fatty acids, ketones, and triglycerides (Paparelli et al. 2013; Pedrotti et al. 2018). Moreover, changes in metabolite concentrations can impose feedback regulation on cellular signaling pathways, further influencing the transcription and translation processes (Kim and Hoxhaj 2022).

As metabolites are key factors in sensing environmental changes, their regulatory mechanisms within organisms are a focal point in the field of life sciences (Li et al. 2024a). In-depth analysis of metabolic networks and signal transduction pathways has shown that metabolites not only provide energy directly, but they also activate or inhibit specific signaling pathways through their interactions with various cell receptors. In addition, metabolites indirectly regulate gene expression by altering cell membrane potential, influencing enzyme activities, or acting as secondary messengers (Vriet et al. 2015). For example, ABA (abscisic acid), a metabolite of sesquiterpenes, functions as a stress-signaling molecule that regulates multiple metabolic pathways, including phenylpropanoid, amino acid, and lipid metabolism, to enhance plant resilience to drought, cold, and other environmental stresses, thereby maintaining plant growth and development (Li et al. 2024a). Studying the functions and regulatory mechanisms of metabolites provides insight into how organisms adapt to changing natural environments. In addition, such analysis provides a theoretical basis and technical support for developing new biomaterials, designing intelligent biological systems, and improving agricultural production (Li et al. 2024b).

It is estimated that over 200,000 metabolites are present in plants, with any single plant species potentially containing 7000–15,000 different metabolites (Fernie and Tohge 2017; Sulpice and McKeown 2015). These metabolites exhibit a high degree of diversity in both structure and function, and their distribution and abundance within the plant are finely regulated across different plant varieties, growth conditions, and stress responses. Plant metabolomics, an important branch of systems biology, focuses on studying the types, quantities, and interrelationships of all metabolites within plants. This field is important for exploring biological processes such as plant growth and development, environmental adaptation, and disease resistance.

The diversity of plant metabolites, the complexity of metabolic pathways, and the wide variation in metabolite levels pose a series of challenges to plant metabolomics research. To address these challenges, researchers have developed various strategies and techniques for separating complex mixtures of metabolites, improving the detection sensitivity of low-abundance metabolites, and simultaneously detecting and quantifying thousands of metabolites for high-throughput analysis (Fernie and Tohge 2017; Sulpice and McKeown 2015). Additionally, high-performance liquid chromatography (HPLC), coupled with a variety of detectors including ultraviolet (UV), photodiode array detection (DAD), fluorescence detection (FD), electrochemical detection (ECD), refractive index detection (RID), flame ionization detection (FID) and charged aerosol detection (CAD), is a powerful and versatile technique for separating plant metabolites in complex matrices (Wolfender 2009).

## Mass spectrometry-based workflow and strategies for plant metabolomics

Plant metabolomics is a rapidly growing field in plant science and systems biology that involves the comprehensive analysis of the types, quantities, and functions of small-molecule natural products in plant tissues and cells, providing a comprehensive understanding of the metabolic profiles of biological systems. Over the past decade, advanced detection technologies have been widely used in plant metabolomics research, among which MS, known for its high sensitivity, high throughput, and exceptional accuracy, has become the tool of choice for plant metabolomics research (Yan et al. 2022). When conducting large-scale studies of highly complex plant metabolites, strategies that combine various separation techniques with MS are typically employed, including gas chromatography–mass spectrometry (GC–MS), liquid chromatography–mass spectrometry (LC–MS), nuclear magnetic resonance (NMR), matrix-assisted laser desorption/ionization (MALDI), capillary-based MS, and other MS-based technologies (Ali et al. 2019; Yu et al. 2023). GC–MS is utilized for the quantitative and qualitative analysis of volatile and thermally stable compounds, whereas LC–MS is capable of analyzing non-volatile or thermally labile high molecular weight compounds that are not amenable to GC–MS analysis, making it an ideal choice for studying complex biological matrices due to its efficiency, simplicity, and robustness (Plumb et al. 2023).

Spatial metabolomics, based on MS imaging, is employed for the precise localization of metabolite distribution in plant tissues (Jing et al. 2024). Mass analyzers with varying resolution capabilities are also employed in metabolomics. These include ultrahigh-resolution and high-resolution mass spectrometers, such as Fourier transform ion cyclotron resonance mass spectrometers (FT-ICR-MS) (Chavez et al. 2022), Orbitrap mass spectrometers, and multi-channel Time-of-flight mass spectrometers (TOF–MS), as well as low-resolution instruments like ion traps (linear and three-dimensional quadrupoles) and single quadrupoles (Table 1). Each of these mass spectrometry analyzers has its own advantages and limitations.

**Table 1:** Comparison of different platforms used in plant metabolomics

Injection mode	Ion source	Resolution	Advantages	Disadvantages	Application	Mass spectrometer	Instrument brand
LC	ESI/APCI	High resolution	High resolution, high accuracy, wide range of applications	Low sensitivity, complex data processing, no standard commercially available metabolic database	Identification of unknown metabolite signals and construction of metabolic databases	Q-TOF	Agilent 6530 Q-TOF LC/MS
						Q-Orbitrap	Thermo Scientific™ Q Exactive™
		Low resolution	High sensitivity, high specificity, enables simultaneous quantification of multiple metabolites	Low resolution, difficult to identify unknown metabolites	Quantitative detection of metabolites	QQQ	Agilent 6495D TripleQuadrupole
						Q-Trap	AB-Sciex 6500Q-trap
GC	EI	High resolution	Uses standard commercial metabolic databases	Complex sample pre-treatment	Identification of unknown volatile metaboles	Q-TOF	Agilent 7250 GC/Q-TOF
		Low resolution	High sensitivity and quantitative accuracy	Low resolution and complex data processing	Quantification of volatile metabolites	Q	Agilent 5977C GC/MSD
Infusion		High resolution	High resolution, high accuracy	Complicated operation and expensive instruments	Identification of unknown metabolites	FTICR-MS	Bruker Daltonik 7T solariX 2XR
			High reproducibility, provides complete structural information	Low sensitivity and lack of a data analysis platform	Obtaining complete structural information on metabolites	NMR	Bruker Daltonik DNP-NMR

The selection of a specific mass spectrometry platform for metabolomics research depends on factors such as the objectives of the study, the efficiency of the platform, and instrument costs. The analysis of metabolites with diverse physicochemical properties cannot be performed using a single stationary phase. Different ion sources have varying ionization efficiencies and characteristics, making them suitable for analyzing different types of samples. The most common ion sources include electron impact ionization (EI), chemical ionization (CI), field ionization/desorption (FI/FD), fast atom bombardment (FAB), atmospheric pressure chemical ionization (APCI), electrospray ionization (ESI), and matrix-assisted laser desorption/ionization (MALDI). LC–MS primarily utilizes ESI and APCI, whereas GC–MS mainly employs EI and CI, and MALDI is widely used for mass spectrometry imaging (MSI) of single cells and tissues (Yin et al. 2024).

**Fig. 1:**
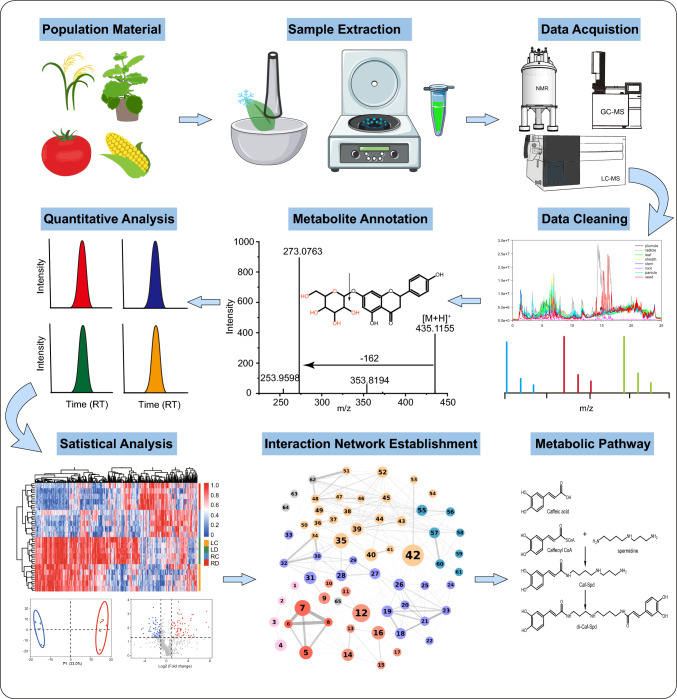
Diagram of the workflow used for metabolomics studies. The workflow of metabolomics studies includes (1) sample collection; (2) sample extraction; (3) data acquisition using LC–MS, NMR, GC–MS, or other methods; (4) data cleaning, including noise reduction, outlier removal, normalization, and other types of analysis; (5) metabolite annotation; (6) quantitative analysis; (7) statistical analysis, including correlation analysis, PCA, KEGG pathway enrichment analysis, and other types of statistical analysis; (8) establishing an interaction network; and (9) metabolic pathway analysis

The basic workflow of plant metabolomics studies includes sample preparation, metabolite extraction and detection, and data processing and analysis (Fig. 1) (Cambiaghi et al. 2017). During metabolomics sample preparation, it is crucial to perform parallel measurements on samples from different individuals, ensuring that biological replicates are properly sampled (Sumner et al. 2007). Additionally, unwanted components such as soil particles must be removed before collecting fresh samples, and freezing methods (such as using dry ice or liquid nitrogen) should be employed during sampling to prevent enzyme-induced metabolic changes. Due to the chemical diversity of metabolites, a single solvent extraction cannot fully capture the entire metabolome. Therefore, after sample collection, different solvents and extraction methods must be chosen and/or combined to extract various metabolites. Polar and semi-polar metabolites can be extracted using water-soluble solvents (e.g., aqueous alcohol solutions), while lipids are better extracted using more lipophilic solvents. A common method is liquid–liquid extraction with chloroform/methanol mixtures in varying ratios to target specific metabolites (Salem et al. 2017). Furthermore, for low-abundance metabolites, single or multi-step solid-phase extraction (SPE), or vacuum concentration and solvent evaporation can be used to concentrate and enrich the extracts (Roessner and Dias 2013). Mass spectrometry techniques and scanning modes are chosen based on the physicochemical properties of plant metabolites, and the resulting data undergo bioinformatics analyses, including peak detection, metabolite identification, quantitative assessment, and metabolic pathway reconstruction. These analyses help reveal dynamic changes in metabolites during plant growth and environmental responses, shedding light on the regulatory mechanisms within metabolic networks (Alseekh et al. 2021a; Shen et al. 2023b).

Due to the diversity and complexity of plant metabolites and different research objectives, plant metabolomics studies require different research strategies, including non-targeted metabolomics, targeted metabolomics, semi-targeted metabolomics, and pseudo-targeted metabolomics (Table 2) (Amer et al. 2023). Non-targeted metabolomics and targeted metabolomics are the classic strategies employed to detect plant metabolites. Non-targeted metabolomics is usually employed when the metabolites are uncertain or unknown (Chen et al. 2021; Naz et al. 2014), as it provides wide coverage and the possibility of discovering new metabolites. Targeted metabolomics, with its high sensitivity and reproducibility, is generally used when the target metabolites are well characterized, or the metabolites must be analyzed in a quantitative manner (Johnson et al. 2016). Phytohormones are important small-molecule compounds present in plants at ultralow levels. A series of targeted metabolomics-based methods were developed for metabolite detection using trace amounts of fresh plant material (< 100 mg) (Xin et al. 2020). These techniques were successfully employed for the targeted analysis of 101 phytohormones and their derivatives, covering the 10 major classes of phytohormones known to date (strigolactones, brassinosteroids, gibberellins, auxin, ABA, ethylene, jasmonic acid, salicylic acid, cytokinins, and polypeptide hormones) (Simura et al. 2018). A chemical-tag-based semi-annotated metabolomics approach, established by integrating non-targeted and targeted metabolomics, identified 1483 metabolites in wheat (*Triticum aestivum*) leaves with at least one known functional structural group (Zhu et al. 2024).

**Table 2:** Comparison of different strategies for plant metabolomics

Strategy	Instrument platforms	Advantages	Disadvantages	Application	References
Target metabolomics	LRMS MRM (multiple reaction monitoring)	Good quantitative ability	Low coverage for metabolite detection	Detection of target metabolites	Gillet et al. 2012
Untargeted metabolomics	HRMS Full MS Auto MS/MS	Unbiased, high coverage	Low sensitivity and quantitative accuracy	Identification of unknown metabolites	Aicheler et al. 2015; Rakusanova and Cajka 2024; Wang et al. 2016
Semi-target metabolomics	HRMS + LRMS/HRMS full MS/dd‐MS2 MRM	High coverage, good quantitative ability	Requires pure chemical standards and internal standards	Biomarker validation and pathway analysis	Amer et al. 2023
Pseudo-targeted metabolomics	HRMS + LRMS Auto MS/MS d-MRM (dynamic MRM)	Wide range of metabolic detection, wide linear range, high reproducibility	No absolute quantification, dependent on instrument sensitivity	Obtain a complete metabolic profile and discover key metabolites	Lv et al. 2020; Chen et al. 2013
Widely targeted metabolomics	HRMS + LRMS Auto MS/MS MRM-EPI (MRM-enhanced product ion) sMRM (scheduled-MRM)	High coverage, high throughput, good reproducibility	Time-consuming processing of large amounts of data	Expansion of metabolic synthesis pathways and discovery of key metabolites	Chen et al. 2014; Chen et al. 2013; Zhu et al. 2018
Widely targeted metabolite modificomics	HRMS + LRMS NL-EPI (neutral loss-enhanced product ion) full MS/dd‐MS2 sMRM	Helps resolve modified metabolites	Heavy metabolic data with redundant metabolic signals	Identification of metabolite modifications and expansion	Yang et al. 2024

To study fungal-induced defense responses in maize (*Zea mays*), non-targeted and targeted metabolomics were employed to identify and quantify flavonoids in leaves of different inbred lines infected with southern leaf blight (*Bipolaris maydis*). The authors detected a particularly high abundance of *O*-methylflavonoids, with *O*-dimethyl-2-hydroxynaringenin being the most abundant compound (Förster et al. 2022). However, with non-targeted metabolomics, it is difficult to accurately quantify metabolites and to detect metabolites present at ultralow levels, whereas with targeted metabolomics methods, the coverage is low (Chen et al. 2023; Simura et al. 2018; Xin et al. 2020). The shortcomings of these two methods limit their use for in-depth plant metabolomics studies.

Widely targeted metabolomics analysis methods, based on triple quadrupole linear ion trap mass spectrometry, have been developed to detect thousands of metabolites sensitively and accurately, enabling high-throughput detection and quantification (Chen et al. 2013). A metabolic approach combining glycosides-specific metabolomics and the use of isotope-labeled precursors can efficiently and accurately identify metabolites of glycosylation in plants (Wu et al. 2022). To further explore possible novel or unknown types of metabolic modifications in plants, a widely targeted metabolite "modificomics" strategy, based on triple quadrupole linear ion trap mass spectrometry and high-resolution mass spectrometry, has been developed for the detection and discovery of a variety of potentially modified metabolites in plants. This strategy has greatly improved the sensitivity of detection and the efficiency of identification of modified metabolites (Yang et al. 2024). A comprehensive analysis of glucosinolates (GLSs) in *Eutrema yunnanense* was carried out using ultrahigh-pressure liquid chromatography coupled with Orbitrap high-resolution mass spectrometry (UHPLC-Orbitrap/HRMS) in the intelligent AcquireX deep scan mode. The authors identified or characterized 175 GLSs, including 52 potentially novel compounds, among which 37 malonylated GLSs were detected for the first time (Geng et al. 2021).

## Analysis of plant metabolomics data and challenges in the omics era

With the improvement of mass spectrometry technology, the throughput and efficiency of metabolomic detection techniques have been rapidly improving. The amount of metabolomic data produced using GC–MS and LC–MS coupling technologies has increased dramatically, and metabolomics research has entered the era of big data. The in-depth analysis of metabolomics data and its integration with genomic, transcriptomic, epigenomic, and other omics data is an important research direction in plant metabolomics. How to manage, analyze, integrate, apply, and share these metabolic data has become one of the biggest challenges in metabolomics.

Metabolomics currently faces three major issues. The first issue is the management and storage of metabolic data. Plants produce a wide variety of metabolites, and different extraction methods and detection instruments may produce data in different formats. The lack of uniform standards makes data integration and sharing difficult. Enormous amounts of metabolomics data are increasingly being generated, requiring effective storage solutions to ensure the long-term preservation and rapid retrieval of these data (MontenegroBurke et al. 2017).

The second issue is the difficulty of analyzing metabolic data and performing integrated analysis (Cambiaghi et al. 2017). Raw mass spectrometry data require preprocessing steps such as baseline correction, peak extraction, and denoising; the accuracy and consistency of these steps directly affect the results of subsequent analysis. Algorithms and software tools for metabolomic data analysis must be developed and optimized to improve the accuracy and efficiency of analysis. A series of data processing methods and software tools have been developed for different metabolomics research strategies and research purposes, such as XCMS (Smith et al. 2006), MS-DIAL (Tsugawa et al. 2020), and MZmine (Schmid et al. 2023) for raw data processing; GNPS (Nothias et al. 2020), SIRIUS (Duhrkop et al. 2019), and MS-FINDER (Mallmann et al. 2023) for metabolite identification; and MetScape (Gao et al. 2010) and Mummichog for functional interpretation (Pang et al. 2024). For example, MS1- and MS2-based analysis strategies for untargeted metabolomics lead to low rates of metabolite identification. A molecular formula-oriented, peak detection-free approach, called HERMES, uses raw LC/MS1 information to optimize MS2 acquisition, improving mass spectral similarity scores and identification rates (Gine et al. 2021). Qualitative (identification) and quantitative (measurement of concentration) analyses of metabolites remain a challenge. Meanwhile, metabolomic data must be integrated with genomic, transcriptomic, proteomic, and other omics data to reveal more comprehensive biological mechanisms.

The third issue is determining how to establish a universal metabolic database. Commonly used metabolomics databases can be divided into four categories: (i) metabolic pathway databases, such as Kyoto Encyclopedia of Genes and Genomes (KEGG) and MetaCyc; (ii) spectral databases, such as METLIN, MassBank, and GNPS; (iii) biological metabolomic databases, such as the Human Metabolome Database (HMDB) and Yeast Metabolome Database (YMDB); and (iv) metabolic data management and public archive databases, including MetaboLights and ProteomeXchange (Nothias et al. 2020; Shi et al. 2019; Tian et al. 2023). Existing metabolic databases might not be comprehensive enough, as there is a need for high-quality annotated information on metabolites, including their chemical structures, functions, and metabolic pathways. The Plant Metabolome Hub (PMhub) is a metabolic database of 188,837 plant metabolites that provides a vast amount of detailed information on their reference spectra, genetic basis, chemical reactions, metabolic pathways, and biological functions. PMhub brings together the corresponding genomic and/or transcriptomic information, allowing a variety of methods to be used for the comprehensive genetic analysis of metabolites (Tian et al. 2023).

Metabolic databases must be regularly updated to reflect the latest research findings while ensuring the quality and reliability of the data. Ensuring the openness and ease of use of databases makes it easy for researchers to access and utilize the data. By addressing these issues, metabolomics data can be better managed, analyzed, and integrated, thus promoting the development of plant metabolomics research and increasing its application value for the genetic improvement of crops and for biomedical research (Li et al. 2023a; Shen et al. 2023a; Wang et al. 2024).

## Integrating multi-omics data to resolve molecular mechanisms of plant metabolic diversity

Metabolomics offers a snapshot of the physiological state of an organism or cell at a specific moment. When integrated with genomics, transcriptomics, and proteomics data, the data can be mutually validated and supplemented, leading to a comprehensive understanding of gene regulatory networks, cell signaling pathways, and protein interaction networks by linking genotypes to phenotypes (Moco et al. 2007; Zhao et al. 2024). In recent years, the combined use of metabolomics with other omics technologies has led to significant advances in life sciences, providing deeper and more holistic insights into the mechanisms and functions of biological systems (Fig. 1).

### Unveiling the holistic nature of biological systems

Metabolomics combined with other omics approaches can provide researchers with comprehensive biological information, offering a panoramic understanding of biological systems from the genome level to the metabolite level (Tolani et al. 2021). Metabolome genome-wide association study (mGWAS) examines genotype–phenotype associations to identify genetic variations linked to complex traits, often revealing multiple candidate loci or genes (Shen et al. 2023b). Advances in high-throughput sequencing have led to the identification of thousands of candidate loci or genes by integrating metabolomics and genetic analysis (Lewin et al. 2018). The amino acid biosynthetic and catabolic pathways in maize kernels were reconstructed based on candidate genes identified by mGWAS of amino acid levels in mature grains of a maize recombinant inbred line population. This analysis provided insights into the genetic basis of amino acid biosynthesis in maize kernels, which could facilitate marker-based breeding for maize with high-quality proteins (Deng et al. 2017).

Multi-omics analysis based on metabolomics can also offer insight into crop domestication. For instance, Zhu et al. revealed changes in nutrient and flavor metabolites in tomato (*Solanum lycopersicum*) fruits during domestication through “metabolomics–genomics–transcriptomics” multi-omics data analysis and identified key genetic loci regulating these metabolites (Zhu et al. 2018). Similarly, Li et al. generated and analyzed a dataset comprising the genomes, transcriptomes, metabolomes, and anti-inflammatory indices of 398 foxtail millet germplasms, elucidating the evolutionary history and metabolite characteristics of this species (Li et al. 2022b). Additionally, since metabolites play crucial roles in plant responses to stress, mGWAS integrating genotypes and metabolite data can be used to identify key genes related to plant-stress resistance. In an mGWAS of flavonoid metabolism, a leucine-rich repeat receptor kinase and a transcription factor gene were identified, cloned, and functionally characterized; these genes positively regulate flavonoid metabolism in rice (*Oryza sativa*), significantly improving tolerance to UV-B stress (Zhang et al. 2022).

### Deciphering regulatory mechanisms of metabolic networks and plant evolution

Metabolomics can be combined with other omics technologies to reveal the regulatory mechanisms of metabolic networks (Lin et al. 2022). By integrating genomics and transcriptomics data, key genes regulating metabolic processes and the underlying pathways can be identified, offering insights into the regulation of metabolic processes (Fernie et al. 2004; Lau and Sattely 2015; Lu et al. 2023). GWAS can identify single-nucleotide polymorphisms (SNPs) associated with complex traits and reveal the roles of key transcription factors in regulating these traits. For example, SlERF.H6 was identified based on mGWAS of steroidal glycoalkaloids (SGA) levels in tomato. This transcription factor directly regulates SGA biosynthetic genes and mediates gibberellin and ethylene signaling (Hao et al. 2023). In another example, a metabolite–SNP–gene interaction network constructed in citrus identified CgMYB1 as a key transcription factor that positively regulates phenylpropanoid metabolism (Wang et al. 2024).

In recent years, the combination of metabolomics with epigenomics and other tools has provided a more comprehensive understanding of plant metabolic systems. Integrative analysis of multi-omics data, including DNA methylation, transcriptomics, and metabolomics data, unraveled the mechanisms for species formation and ecological stress adaptation in wild barley populations from two geographically similar, but ecologically different regions (Cai et al. 2021). Lu et al. proposed the concept of a “epigenome–metabolome–epigenome” cross-level cascade response, revealing the central role of glycolytic metabolism-driven histone acetylation and lactylation modifications in connecting early and late epigenetic events (Lu et al. 2023). Furthermore, by analyzing wild, local, and cultivated tomato varieties using whole-genome bisulfite sequencing (WGBS), RNA sequencing (RNA-seq), and metabolomics, Guo et al. (2023) elucidated the relationship between metabolic diversity and changes in DNA methylation during breeding, constructed a multi-omics correlation network, and revealed the biosynthetic pathways of polyphenols in tomato (Guo et al. 2023).

Metabolites play important roles in plant evolution and environmental adaptation, and plant metabolomics provides new insights into plant evolution by comprehensively analyzing the types and levels of metabolites in plants. Xu et al. (2019) performed metabolomic analysis of teosinte, tropical maize, and temperate maize, representing the major stages of maize evolution. These authors identified 461 metabolites exhibiting significant differences due to selection, with alkaloids, terpenoids, and lipids specifically targeted in the divergence between teosinte and tropical maize, and benzoxazinoids in the divergence between tropical and temperate maize. By integrating their metabolomic data with transcriptomic data, these authors identified candidate genes responsible for metabolic differences among the maize accessions and maize relatives in their study, providing important insights into the metabolic changes associated with maize domestication (Xu et al. 2019).

Schneider et al. (2021) analyzed the compositions of secondary metabolites in specific plant organs by non-target metabolomics and molecular network analysis and compared multiple dimensions of phytochemical diversity across organs. These authors determined that plant organ characteristics significantly influence the compositions of secondary metabolites, both independently of and together with species characteristics. In addition, by performing comparative analysis of plant metabolic biosynthesis networks, the origin of alien plant metabolic pathways and their related enzymology were identified in kava and grass, respectively (Lui et al. 2023; Pluskal et al. 2019).

### Exploring metabolite biosynthesis pathways

Combining metabolome and transcriptome data can reveal the functions of metabolic pathways by identifying potential biosynthetic genes across tissues. Plant metabolic gene clusters (i.e., closely linked, co-regulated non-homologous genes in the same biosynthetic pathway) serve as valuable tools for characterizing the biosynthetic pathways. The development of multi-omics approaches (combining two or more techniques, including genomics, transcriptomics, metabolomics, or epigenomics) provides new strategies and opportunities for the discovery of natural product pathways (Du et al. 2024; Ma and Qi 2021; Nett et al. 2020). Jeon et al. (2020) identified a pathogen-responsive gene cluster responsible for falcarindiol biosynthesis in tomato by combining metabolomics and RNA sequencing analysis (Jeon et al. 2020). A metabolic gene cluster for the biosynthesis of sesquiterpene with antimicrobial activity specific to *indica* rice was identified using the same approach (Zhan et al. 2020). In addition, two phenylalanine gene clusters involved in disease resistance in rice were identified based on mGWAS (Shen et al. 2021). Similarly, two tomato phenylamine biosynthetic gene clusters were elucidated revealing the role of phenylamines in reducing drought tolerance during tomato domestication and improvement (Cao et al. 2024).

By combining metabolomics and multi-omics, metabolite–gene associations can be analyzed at the whole-genome level, identifying genetic variations related to specific metabolite concentrations or metabolic pathways, leading to the identification of enzymes directly affecting the production, transformation, or degradation of metabolites (Zhu et al. 2023). Tohge et al. (2020) conducted a metabolomics study on different tomato varieties. Using these data, combined with publicly available RNA sequencing data, these authors constructed a species-specific metabolic network for polyphenol metabolism, establishing a framework for exploring polyphenol biosynthesis, annotating metabolites, and examining the expression levels of genes involved in the flavonoid pathway. Furthermore, the combination of multi-omic techniques identified a large number of new metabolic regulatory genes encoding proteins that regulate the synthesis or metabolism of metabolites by influencing enzyme activity or gene expression levels. The BAHD-type acyltransferases and 2-oxoglutarate-dependent dioxygenases were identified using metabolome and transcriptome datasets, further refining the SGA biosynthetic pathways (Cardenas et al. 2019; Sonawane et al. 2023a, 2023b). Overall, the combination of metabolomics with other omics technologies provides a more comprehensive, in-depth perspective on plant systems, offering an important foundation for better understanding the adaptation of plants to different environments.

## Plant metabolomics contributes to nutritional quality and stress tolerance in crops

**Fig. 2:**
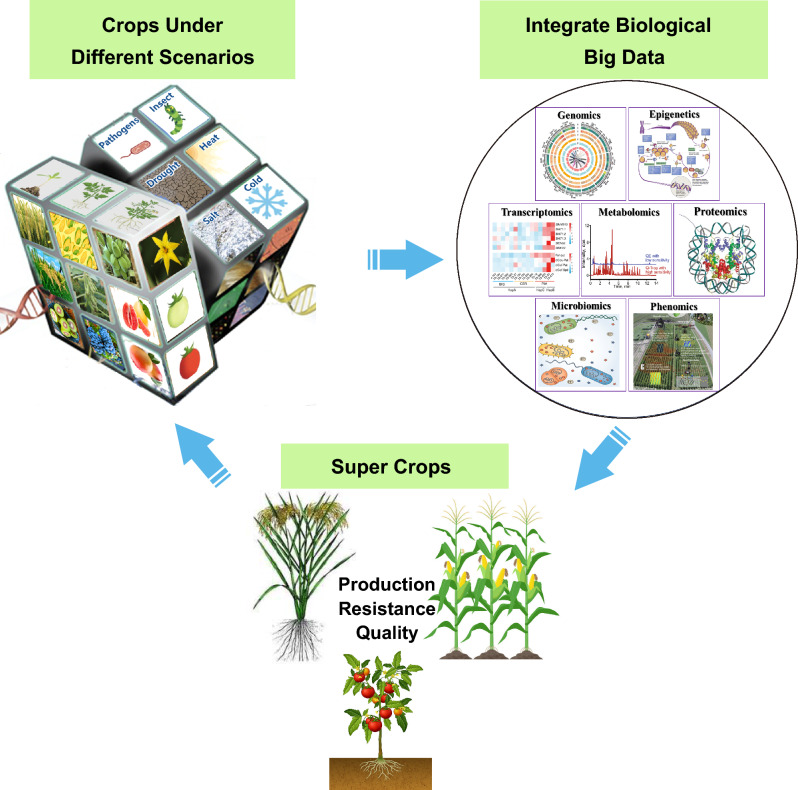
Strategy for breeding superior crops based on the results of multi-omics analysis. Genomics, epigenetics, transcriptomics, proteomics, microbiomics, and phenomics data from different species, tissues, developmental stages, and stress conditions can be integrated for the efficient aggregation of traits for improved nutritional quality and stress resistance in crops

Plants exhibit enormous chemical diversity and metabolic complexity. Metabolites play crucial roles in various aspects of plant biology such as growth, development, stress responses, and nutritional quality. Analyzing metabolic data is important for understanding and improving the yield, quality, and resistance traits of crops (Alseekh et al. 2021b). Through comprehensive analysis and an in-depth understanding of plant metabolites, the molecular mechanisms affecting the nutritional value and resistance of crops can be revealed, thereby guiding breeding and agricultural management practices, ultimately resulting in improved crop quality and yield (Fig. 2).

Metabolomics technology enables the precise identification and quantitative analysis of various metabolites in plants, such as sugars, lipids, amino acids, and vitamins, which directly determine the nutritional value of crops (Fang et al. 2018; Wang et al. 2019). During the domestication and improvement of crops, metabolites undergo significant changes. For example, cucurbitacins and flavonoids have been negatively selected in melons, and the ratios of sugars, carotenoids, citric acid, and malate have increased (Yuan et al. 2023). Changes in the contents of steroidal alkaloids and cucurbitacin in potatoes (*Solanum tuberosum*), tomatoes, and cucumbers have made these foods less bitter (Itkin et al. 2013; Zhu et al. 2018). Metabolomics analysis of different crop varieties or transgenic crops can lead to the identification of metabolic markers related to nutritional quality, which can be used to screen for superior varieties with high nutritional quality. For example, analysis of metabolomic differences between high oleic soybean (*Glycine max*) varieties and conventional varieties could be used to guide breeding efforts (Yun et al. 2020). More recently, a kiwifruit metabolic regulatory network was identified by analyzing the metabolic landscapes of this crop at 11 different stages of development and ripening, providing a valuable resource for the designed improvement of kiwifruit quality (Shu et al. 2023). Metabolome analysis of pulp samples from 292 accessions of wild and cultivated apple revealed significant differences in flavor compounds and quality traits, including tannins, flavonoids, phenolic acids, organic acid lipids, and phytohormones (Lin et al. 2023). The key genes regulating the biosynthesis of these substances were identified through mGWAS, providing powerful data for improving the flavor, nutritional value, and quality of apples in the future.

Plants have the remarkable ability to synthesize a vast array of metabolites that differ in chemical complexity and biological activity, playing indispensable roles in defense against both biotic and abiotic stress (Garagounis et al. 2021). Metabolomics can identify resistance-related metabolites produced by plants in the face of biotic or abiotic stress (e.g., pests and diseases, drought, salinity, and low temperature), which may include secondary metabolites, antioxidants, and other defense compounds. Sakuranetin, a flavonoid metabolite regulated by jasmonic acid signaling in rice, reduces the number of yeast like symbionts (YLS) and cholesterol accumulation in brown planthoppers, thereby enhancing the resistance of rice to these insect pests (Liu et al. 2023). Rice also produces bayogenin 3-*O*-cellobioside, which inhibits the germination of rice blast fungus conidia and attachment cell formation, enhancing resistance to rice blast disease (Norvienyeku et al. 2021). Eriodictyol, which is derived from naringenin in the phenylpropanoid pathway, inhibits the growth of rice blast fungus and enhances plant resistance to brown planthopper (Chen et al. 2022). The metabolic profiling of 60 rice varieties of varying drought tolerance (including 30 *indica* and 30 *japonica* varieties) from a core germplasm panel under drought stress and normal irrigation conditions identified 233 drought-responsive metabolites, including phenylamines, polyamines, phytohormones, and flavonoids (Guo et al. 2024). Wang et al. (2023) performed metabolomic analysis of the quality differences and physiological characteristics of rice cultivated under drought vs. flooded conditions. Based on the results, these authors proposed that increasing the water supply to appropriate levels, reducing nitrogen fertilization, and increasing silicon fertilization can improve the quality of dryland rice, including its taste.

Metabolomics, in combination with genomics, transcriptomics, and other omics technologies, can be used to identify key genes and metabolic pathways related to plant resistance, analyze the metabolic response mechanisms of plants under stress conditions, and identify metabolic pathways and regulatory networks related to resistance, thereby providing detailed guidance for improving crop resistance via breeding. For example, researchers identified two biosynthetic gene clusters (BGC7 and BGC11), containing 12 genes, which promote phenolamide biosynthesis and enhance drought tolerance in tomato (Cao et al. 2024). The application of sterol, fucosterol, and soyasaponin II significantly improved drought tolerance in soybean, wheat, foxtail millet, and maize (Yu et al. 2024). The UDP-glucosyltransferase GSA1 (GRAIN SIZE AND ABIOTIC STRESS TOLERANCE 1) synergistically regulates grain size and stress resistance by modulating the direction of metabolic flux in rice under drought stress (Dong et al. 2020). Metabolites exhibit both spatiotemporal-specific accumulation and diversity in plants under stress. Metabolomics helps uncover the biochemical basis of plant phenotypes, and the reshaping of the metabolome under stress conditions largely reflects the plant’s response to stress. The analysis of metabolic mechanisms lays the foundation for breeding highly resistant, high-quality crops.

## Hurdles, opportunities, and future perspectives

Plant metabolomics has made considerable progress in recent years, but still faces significant challenges and limitations. Advances in mass spectrometry and nuclear magnetic resonance technology have greatly improved the sensitivity of plant metabolomics. However, due to the difficulty in separating, identifying, and quantifying polar compounds and low-abundance metabolites, as well as the influence of sample preparation, analytical techniques, and data processing, it is challenging to simultaneously detect low and high-abundance metabolites in the same sample. Additionally, with the development of computational biology and bioinformatics, the interpretation and analysis of plant metabolomics data have become more efficient and accurate. However, challenges still exist in the interpretation of plant metabolomics data, especially in terms of integrating and explaining large-scale data. Currently, data interpretation is hampered by both biological complexity and data processing limits, leading to the underutilization of some information (Tolani et al. 2021). Algorithms and machine learning can enhance the interpretation of metabolomics data by uncovering hidden patterns and associations, while also assisting in feature selection and dimensionality reduction to identify the most important variables (Chen et al. 2024; Ma and Qi 2021).

Integrating multiple omics tools is an inevitable trend for comprehensively investigating biological systems. Integrative analysis of plant metabolomics data with other omics data, including genomics, transcriptomics, and proteomics data, provides researchers with a comprehensive platform of germplasm information and multi-omics data, providing better access to public resources for research and breeding (Li et al. 2022a, 2023b; Yang et al. 2023). Furthermore, combining metabolomics with phenomics, epigenomics, ionomics, and microbiomics approaches allows for more comprehensive monitoring of plant metabolic systems (Huang et al. 2019; VenegasMolina et al. 2021). However, integrating multi-omics approaches faces challenges, such as data inconsistency, the need for standardization, and the need for integrated analysis methods. Due to continuous improvements in biotechnology, new detection techniques and methods are constantly emerging, providing more possibilities for in-depth research on plant metabolomics. For example, gene editing technologies such as CRISPR-Cas9 can be used to precisely modify plant metabolic pathways, which helps reveal their regulatory mechanisms and functions. In parallel, high-throughput sequencing technology can quickly and accurately produce large amounts of genetic and transcriptomic data, providing more data support for the integration of metabolomics with other omics technologies.

Since its emergence and rapid development, metabolomics has been widely utilized across various fields of life sciences. Metabolomic analysis can provide a comprehensive view of the metabolic network and its fluctuations within an organism, highlighting differences in metabolic profiles across species, between different tissues of the same species, and within the same tissue under varying stress conditions. Integrating metabolomics with other omics approaches, such as genomics, transcriptomics, proteomics, and phenomics, offers valuable insights into the plant metabolome, metabolic pathways, and the biochemical and genetic basis of stress responses. Metabolomics technology enables the precise detection of metabolites in different plants, facilitating the analysis of metabolic pathways, with a focus on the diversity of phytonutrients and their genetic underpinnings. Additionally, nutrient-related molecular markers could be utilized in crop breeding, to explore the important roles of metabolite biosynthesis, and in metabolomics-assisted breeding to improve the nutritional quality of crops in the future. Metabolomics-driven breeding could provide crops with added nutritional value and enrich the genetic resources available for breeding cultivated crop varieties. Moreover, integrating metabolomics with other omics tools, along with reverse genetics tools, represents a key approach for quickly identifying resistance genes involved in plant–environment interactions and breeding resistant crop varieties. Looking ahead, advancements in analytical technology platforms, more refined isolation and detection methods, powerful data processing tools, and comprehensive metabolome databases are expected to amplify the impact of metabolomics in biology, agriculture, medicine, and environmental research.

## Conclusions

In this era of multi-omics big data, the integration of plant metabolomics with other omics techniques has become an important trend. Through the integrated analysis of multi-omics data, comprehensive plant metabolomics platforms provide genetic information to promote resource sharing and utilization. At the same time, combining metabolomics with other omics techniques allows for more comprehensive monitoring of plant metabolic systems. However, the integration of multi-omics techniques also faces challenges, such as difficulties in maintaining data consistency and the need for standardization and integrated analysis methods. Further improving detection technology, developing more efficient data processing methods, and strengthening integration and collaborative analysis among different omics data can lead to a deeper understanding and the increased utilization of plant metabolomics. Overall, the application prospects of plant metabolomics in the era of multi-omics big data are vast, but there are many challenges. By continuously optimizing the technology and strengthening data integration and analytical collaboration, the outcome should be a better understanding and utilization of plant metabolomics data, providing an enhanced theoretical and practical foundation for future research.

## Data Availability

Data sharing is not applicable to this article as no datasets were generated or analyzed in the study.
